# Urinary Prothrombin Fragment 1+2 in relation to Development of Non-Symptomatic and Symptomatic Venous Thromboembolic Events following Total Knee Replacement

**DOI:** 10.1155/2011/150750

**Published:** 2011-05-03

**Authors:** Lars C. Borris, Morten Breindahl, Michael R. Lassen, Ákos F. Pap

**Affiliations:** ^1^Department of Orthopaedics, Århus University Hospital, Nørrebrogade 44, 8000 Århus C, Denmark; ^2^Neonatalklinikken GN5023, Rigshospitalet, Blegdamsvej 9, 2100 København, Denmark; ^3^Department of Orthopaedics, Nordsjællands Hospital Hørsholm, Usserød Kongevej 102, 2970 Hørsholm, Denmark; ^4^Bayer HealthCare AG, Aprather Weg 18a, 42096 Wuppertal, Germany

## Abstract

Prothrombin fragment 1+2 is excreted in urine (uF1+2) as a result of *in vivo* thrombin generation and can be a marker of coagulation status after an operative procedure. This study compared uF1+2 levels in patients with symptomatic and non-symptomatic venous thromboembolism (VTE) after total knee replacement (TKR) and in event-free sex- and age-matched controls. Significantly higher median uF1+2 levels were seen in the VTE patients on days 1, 3, and the day of venography (mostly day 7) after TKR compared with controls. The uF1+2 levels tended to be high in some patients with symptomatic VTE; however, the discriminatory efficacy of the test could not be evaluated. In conclusion, this study showed that patients with VTE tend to have significantly higher uF1+2 levels compared with patients without events between days 1 and 7 after TKR surgery. Measurement of uF1+2 could provide a simple, non-invasive clinical test to identify patients at risk of VTE.

## 1. Introduction

Venous thromboembolism (VTE) is a common consequence of orthopaedic surgery and occurs more frequently after total knee replacement (TKR) than after total hip replacement (THR) [1]. Anticoagulation therapy is recommended in the perioperative period, although the optimal duration of treatment and the optimal timing of the first dose remain unresolved [1]. Patients at high risk of VTE will benefit from appropriate thromboprophylaxis after hospital discharge [1]. Therefore, a non-invasive clinical test would be a valuable tool for surgeons to identify the TKR patients who are specifically at a risk of venous thromboembolic events.

During the conversion of prothrombin to thrombin, the prothrombin fragment 1+2 (F1+2, molecular weight ~31,000 D [2]) is formed as a by-product [3]. Therefore, the coagulation status can be determined by measuring F1+2 levels in plasma [4, 5]. Due to its molecular size, F1+2 is excreted in urine (uF1+2). It has previously been reported that measurements of uF1+2 by an enzyme-linked immunosorbent assay (ELISA) can be used to identify patients at risk for symptomatic and asymptomatic VTE and symptomatic arterial vascular events after THR [6, 7]. Furthermore, in patients who experienced bleeding complications after THR, a significantly less pronounced coagulation response with low median uF1+2 levels was seen in the postoperative period compared with sex- and age-matched control patients and THR patients with venous thromboembolic events [7]. 

The present study was performed to assess whether measurements of uF1+2 would also be useful in patients undergoing TKR to differentiate between patients who are at risk for VTE or bleeding. This study showed that patients with VTE tend to have significantly higher uF1+2 levels when compared with patients without events between days 1 and 7 after TKR surgery.

## 2. Methods

### 2.1. Study Design

This study was conducted in parallel with a phase II investigation of rivaroxaban for the prevention of VTE in patients undergoing primary elective TKR [8]. Detailed inclusion and exclusion criteria have been published previously [8]. Patients received oral rivaroxaban (2.5, 5, 10, 20, or 30 mg twice daily) or subcutaneous enoxaparin (30 mg twice daily) for 5–9 days. Continued thromboprophylaxis after completion of the study period was at the discretion of the investigators. The local ethics committees approved the study, and all patients provided written, informed consent before participation. Mandatory bilateral venography was performed 5–9 days after the operation, most commonly on days 6 (28/142), 7 (57/142), or 8 (36/142). The local radiologists initially assessed the venograms, and an expert adjudication committee subsequently performed a central assessment. Only the results of the central assessments are used in this study. Diagnoses of symptomatic VTE, deep vein thrombosis (DVT), or pulmonary embolism (PE) were confirmed by objective testing.

### 2.2. Patients and Controls

All patients with a venous thromboembolic event, symptomatic or non-symptomatic, were included in the present study. Age- and sex-matched controls, without events either at the local or at the central reading or other vascular events/death, were randomly selected without knowledge of their uF1+2 results. In the case of deaths where vascular involvement could not be ruled out, urine samples were also analysed.

### 2.3. Laboratory Analyses and Urine Sampling

Spot urine samples were collected from patients in the morning before TKR surgery, on days 1 and 3 after surgery, on the day of venography (between day 5 and 9) and at follow-up (day 39). The samples were snap-frozen and stored at −80°C until batchwise analysis after completion of the main study. The analyses were performed by a laboratory technician blinded to the clinical course of the individual patients. Levels of uF1+2 were measured using a commercially available ELISA kit (Enzygnost F1+2, monoclonal, Dade Behring, Marburg, Germany) on a BEP 2000 analyser (Dade Behring, Marburg, Germany). The previously reported cut-off level of uF1+2 between 0.3 and 0.5 nmol/L for symptomatic VTE events [6] was based on analyses with another ELISA kit (micro) from the same manufacturer. According to information supplied from the company, the following equation can be used to convert between the two kits: ELISA 1 (monoclonal) = 0.265 × ELISA 2 (micro)−29.4 in pmol/L. Thus, in this context, a uF1+2 between 0.3 and 0.5 nmol/L is equivalent to uF1+2 between 50 and 103 pmol/L with the new kit.

### 2.4. Statistical Analyses

Matching was done by randomly selecting one patient from the suitable event-free controls for each of the VTE patients. Multiple logistic regression analysis was used to explore the relationship between the incidence of VTE events and uF1+2 levels on the different sampling days, with treatment group as a factor [9–11]. The linear term of uF1+2 levels was included as a covariate in these models. The nonparametric Wilcoxon rank sum test was used to compare the medians of uF1+2 levels between VTE cases and controls on the different sampling days. This method was used because the data were not normally distributed within cases and controls. The results were considered statistically significant if the two-sided *P* value was <.05. The statistical program package SAS (versions 8.2 and 9.1) was used for the statistical evaluation.

## 3. Results and Discussion

Three patients with symptomatic events, two with PE (of which one was fatal on day 41) and one with DVT, were excluded from the analyses because of missing urine samples on all of the days of sampling. This study included 142 patients with a VTE event and 140 control cases. Four patients had PE and 138 had DVT, of which seven were symptomatic and the rest were non-symptomatic. Three deaths occurred, two due to PE and one due to cardiorespiratory failure. The baseline characteristics of the cases and the control group were similar (Table 1), and there was an equal distribution in terms of weight, height, and body mass index. No differences in renal function, as indicated by creatinine clearance, were seen between the two groups. The distribution of patients according to study medication (type and dose) and the median uF1+2 levels on any sampling day are shown in Table 2. In general, the median uF1+2 levels in the rivaroxaban groups were higher on the day of venography in the VTE cases compared with the controls with substantial variation between the different dose regimens, but with no differences seen on the other days. Logistic regression models suggested that there were no differences across the treatment regimens in terms of the uF1+2 levels preoperatively (*P* = .51) or on the day of follow-up (day 39) (*P* = .35). However, on the day of venography, uF1+2 was positively associated with the observed VTE event rate (*P* = .0015), but there was also a residual effect of rivaroxaban versus enoxaparin (odds ratio, 0.40; 95% confidence interval, 0.20–0.76; *P* = .0049). The study was not powered to show any dose–response differences between the rivaroxaban doses (*P* = .18). Due to the non-symptomatic nature of the majority of the VTE events and their unknown clinical importance, it was decided to compare the uF1+2 levels in VTE cases and controls by pooling the different study medications and regimens on the respective days of sampling.

**Figure 1:**
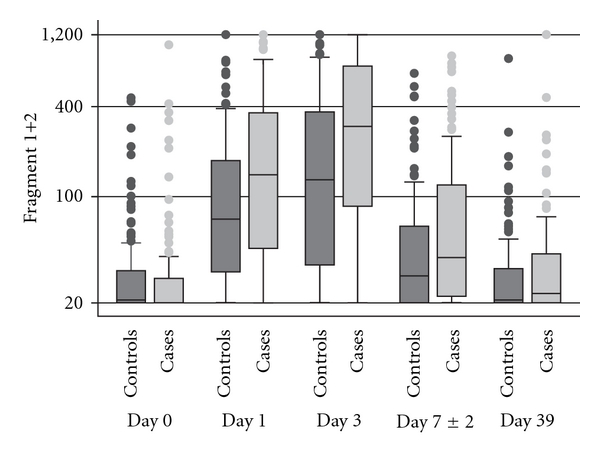
Box-and-whisker plot for the prothrombin fragment 1+2 excreted in urine. Box-and-whisker plot for the prothrombin fragment 1+2 excreted in urine in the venous thromboembolism group (Cases) and the matched, event-free, control group on each sampling day. Prothrombin fragment 1+2 levels (pmol/L) are plotted on a logarithmic scale. Day 0 is the day of total knee replacement. Day 7 ± 2 is the day of venography. Day 39 is the day of follow-up. Boxes are lower and upper quartiles with the median represented as the line in the box. The extremes of the whiskers are the quartiles ± 1.5 times the interquartile range, with outliers given as points.

Table 3 shows the median uF1+2 levels on the respective days of sampling in VTE cases and controls. The uF1+2 levels were not statistically different in the two groups preoperatively and at follow-up (*P* = .44 and *P* = .32, respectively); however, on day 1 and 3 after the operation and on the day of venography, the median uF1+2 level in the VTE patients was significantly higher than the median level in the controls. The day-to-day changes of the median uF1+2 levels observed are shown schematically in Figure 1. The median uF1+2 level increased from the day of surgery reaching a maximum on day 3 in both cases and controls; it then declined, and at follow-up it approximated the preoperative level.

There were 11 symptomatic VTE events in patients with available urine samples on at least one of the sampling days. Urine samples were missing on the day of venography in four patients, two with PE and two with symptomatic DVT.  Table 4 shows the uF1+2 levels preoperatively and on the day of venography (day of urine sampling). In addition, the day and the type of event are shown. There was a tendency towards lower uF1+2 levels when there was a time delay between the day of urine sampling and the day of the event. The variations seen may be due to differences in prophylactic treatment and variations in kidney function. A 71-year-old woman who possibly had a fatal PE on day 14 (but this was never established by objective means) and a 53-year-old woman who developed symptoms of PE on day 23 (her venography was normal on day 8) both had uF1+2 <20 pmol/L on the day of venography. There was no evidence of differences in uF1+2 levels between men and women.

In this study of patients who had undergone TKR, significantly higher uF1+2 levels were observed on day 1 and 3 after the operation and on the day of venography in patients with VTE events (symptomatic and non-symptomatic) compared with age- and sex-matched event-free controls. This indicates that measurement of uF1+2 is also feasible in this orthopaedic patient category.

It is well known that TKR patients differ from THR patients in terms of risk of venous thromboembolic events, type and location of events, and timing of events after surgery [1]. The incidence of DVT after TKR is higher than after THR, but with lower rates of proximal thrombi and symptomatic VTE events, and there is generally an earlier occurrence of postsurgical symptomatic events [12, 13]. These observations could lead to the assumption that the temporal sequence of activation of coagulation after TKR is more intense than after THR and has been explained by the fact that the operative procedure used in knee arthroplasty involves more bone and soft tissue traumatization than the procedure used in hip arthroplasty, and thereby results in more tissue factor release and subsequently in more pronounced thrombin generation. Some of the observed uF1+2 levels in the present study seen in both the VTE patients and the controls seem to support this hypothesis. When comparing observations on day 3 in a parallel and very similar study with THR patients [7], the median uF1+2 levels in that study were 127.3 pmol/L in the VTE patients and 70.6 pmol/L in the controls. This was considerably lower than the levels of 279.6 pmol/L and 130.1 pmol/L, respectively, observed on the same day relative to surgery in the present study. In addition, on the day of venography, the levels were higher after TKR (39.7 pmol/L) than those after THR (30.8 pmol/L) in the VTE patients but at almost the same level in the controls in the present study (30.5 pmol/L) and in the previous study (30.1 pmol/L). It should, however, be noted that some of the venographies were done one day earlier in the present study (60% on days 6 and 7) compared with the THR study (79% on day 7 or later), but this is unlikely to explain the markedly higher uF1+2 levels in the VTE patients. These observations could be an indication of more pronounced coagulation activation after TKR in the first days after surgery.

Unlike in the THR study [7], a significantly higher median uF1+2 level was observed on day 3, but not on the day of venography in VTE patients compared with the event-free controls. Significantly higher uF1+2 levels were observed on days 1 and 3 and the day of venography in the present study, which is also an indication of more pronounced coagulation in these patients. However, the day-to-day changes of the median uF1+2 levels are similar during the time course after TKR, with the maximum elevation seen on day 3 after surgery (Figure 1). This is similar to results reported previously in a study of THR patients [6]. Thus in principle, by using the test described in this paper, it would be possible to identify which patients need continued thromboprophylaxis due to continued coagulation activity, and thus a continued risk of VTE events. In a global registry study, it was reported that 27% of TKR patients did not receive prophylaxis 7 days after surgery, although symptomatic VTE events occurred after discharge in 57% of the patients [13].

Eleven patients in this study developed a symptomatic venous thromboembolic event, most of which occurred early in the postoperative period. On the day of venography, uF1+2 levels in some of these patients did not differ from uF1+2 levels in patients with asymptomatic events. In addition, the uF1+2 levels in some of these patients did not even reach the lower end of the previously presented cut-off interval of 50 pmol/L [7]. An explanation for this could be that the same cut-off cannot be applied to THR and TKR patients. Urine samples from the day of venography were missing in four patients (36%) in this study. In addition, there was a substantial time delay between the day of urine collection and the day of the event, 10 to 15 days, respectively, in two cases (in one the venography was normal and in another an objective diagnosis of PE was never established because the patient died). It was originally planned to have a urine sample on the day of the event; however, this was not done in any of these cases and, therefore, it is not readily possible to assess whether the uF1+2 levels changed, as seen in a previous study after THR [7]. It is, however, recommended that the test is repeated after surgery at preset time intervals, always after cessation of prophylaxis and whenever there is a clinical suspicion of a venous thromboembolic event. This is very easy to do due to the non-invasive character of this test principle.

D-dimer (DD) is often used to rule out the presence of VTE in a patient with clinical symptoms. The diagnostic capacity of DD in the diagnosis of non-symptomatic DVT compared with venography has been studied after THR and TKR. However, the results were disappointing, probably due to the activating effect of surgery *per se*, and the test was not recommended in these patient groups [14, 15].

## 4. Conclusions

Although the results presented hint at the usefulness of the measurement of uF1+2 levels, no firm conclusions can be drawn as to whether measurement of uF1+2 levels can be used routinely to assess the presence of VTE after TKR, except that it seems to be more suited for testing postoperative patients than DD.

## Figures and Tables

**Table 1:** Baseline characteristics of the cases and the sex- and age-matched, event-free control group. *Creatinine clearance for men: (140 − age) × weight / (72 × serum creatinine, mg/dL); creatinine clearance for women: 0.85 × (140 − age) × weight / (72 × serum creatinine, mg/dL).^ †^ *n* = 138. ^‡^ *n* = 137.

	Venous thromboembolic event patients (*n* = 142)	Control patients (*n* = 140)
Males/females, *n* (%)	50/92 (35.2/64.8)	50/90 (35.7/64.3)
Age, years, median (range)	70 (48–85)	69 (48–86)
Height, cm, median (range)	165.1 (142–195.6)	165.0 (140–193)
Weight, kg, median (range)	86.3 (52–124.9)	84.1 (53.3–140.9)
Body mass index, kg/m^2^, median (range)	30.3 (20.8–48.4)	31.0 (19.7–49.5)
Creatinine clearance, mL/min; calculated, median (range)*	92.4 (34.9–224.7)^†^	88.6 (32.0–184.0)^‡^

**Table 2:** Levels of prothrombin fragment 1+2 in urine in relation to treatment regimen. Lower limit of detection 20 pmol/L, upper limit 1,200 pmol/L. bid: twice daily; uF1+2: prothrombin fragment 1+2 in urine; VTE: venous thromboembolism.

Level of uF1+2, pmol/L, median (range)	Rivaroxaban										Enoxaparin	
	2.5 mg bid		5 mg bid		10 mg bid		20 mg bid		30 mg bid		30 mg bid	
	Cases (*n* = 22)	Controls (*n* = 17)	Cases (*n* = 25)	Controls (*n* = 21)	Cases (*n* = 16)	Controls (*n* = 32)	Cases (*n* = 22)	Cases (*n* = 15)	Controls (*n* = 24)	Cases (*n* = 30)	Controls (*n* = 17)
Preoperative	20 (<20–136.5)	27.1 (<20–287.3)	20.5 (20–414.8)	20 (<20–32.8)	20 (<20–1,025)	23 (<20–457.5)	20.1 (<20–68.1)	20 (<20–431.2)	25.6 (<20–323)	20 (<20–127.3)	20 (<20–212.6)	27.9 (<20–190.8)
Day of venography	31.3 (<20–475.7)	29.5 (<20–542)	82.2 (<20–869.1)	24.9 (<20–470.4)	35.5 (<20–780.9)	31.8 (<20–660.2)	43.6 (<20–702.5)	31 (<20–461.5)	50.2 (<20–501.2)	23.3 (<20–212.6)	31.4 (<20–852)	44.6 (<20–243.7)
Day of follow-up	22.2 (<20–74.3)	23.9 (<20–117.4)	22.1 (<20–258.3)	20 (<20–831.8)	23.1 (<20–>1,200)	20 (<20–269.9)	25.9 (<20–460.2)	20.9 (<20–80.6)	24 (<20–152.7)	20.9 (<20–113.5)	20 (<20–192.9)	24.3 (<20–161.7)

**Table 3:** Levels of prothrombin fragment 1+2 excreted in urine in the VTE and the control groups. Values shown are median (lower limit of detection 20 pmol/L, upper limit 1,200 pmol/L). *Wilcoxon test (two-sided). ^†^Day of venography was day 7 ± 2 after surgery. TKR: total knee replacement; VTE: venous thromboembolism.

	Prothrombin fragment 1+2 levels in urine, pmol/L, median (range)		
	VTE group (*n* = 142)	Control group (*n* = 140)	*P* value*
Day of TKR	20 (<20–1,025)	20.7 (<20–457.5)	.45
Day 1 after TKR	141.0 (<20–>1,200)	71.8 (<20–>1,200)	.0032
Day 3 after TKR	295.9 (<20–>1,200)	130.8 (<20–>1,200)	.0002
Day of venography^†^	39.7 (<20–869.1)	30.0 (<20–660.2)	.0083
Follow-up (day 39)	23.0 (<20–>1,200)	20.9 (<20–831.8)	.3445

**Table 4:** Results for patients with symptomatic venous thromboembolic events. Patients with symptomatic venous thromboembolic events, type of event, day of event, day of venography, and levels of prothrombin fragment 1+2 excreted in urine preoperatively and on the day of venography. Lower limit of detection 20 pmol/L, upper limit 1,200 pmol/L. CT: computerized tomography; CUS: compression ultrasound; DVT: deep vein thrombosis; NS: no urine sample; PE: pulmonary embolism; uF1+2: prothrombin fragment 1+2 excreted in urine.

Sex, M/F	Age, years	uF1+2 preoperatively, pmol/L	Day of venography (day of urine sampling)	uF1+2 on day of venography, pmol/L	Type of event	Day of event	Comment
F	71	NS	10	<20	PE	14	Bilateral asymptomatic distal DVT. Not treated. Fatal PE could not be excluded, but was never objectively confirmed
F	53	27	8	<20	PE	23	Planned venography normal
F	68	<20	—	NS	PE	4	PE confirmed by CT
M	62	<20	—	NS	PE	8	PE confirmed by CT
M	51	68.1	8	702.5	DVT	8	Distal DVT. Diagnosed by planned venography
F	73	21.5	6	46.5	DVT	7	Distal DVT. Symptoms appeared on the day after the planned venography
F	55	35.4	5	28.5	DVT	7	Distal DVT. Diagnosed by planned venography
F	80	<20	7	31.6	DVT	7	Distal DVT. Diagnosed by planned venography
F	60	<20	—	NS	DVT	7	Diagnosed by planned venography
F	71	58.7	6	168.2	DVT	16	Proximal DVT. Planned venography normal. CUS diagnosis on the event day
M	62	54.5	—	NS	DVT	5	CUS diagnosis venography not performed
